# Green Shoots in a Barren World: Recent Developments in International Investment Law

**DOI:** 10.1007/s40802-020-00175-2

**Published:** 2020-10-26

**Authors:** Jason Rudall

**Affiliations:** Public International Law, Grotius Centre for International Legal Studies, Leiden University, Leiden, The Netherlands

**Keywords:** International investment law, Environment, Human rights, Treaties, Investor-state dispute settlement

## Abstract

This article begins from the observation that there have been a number of developments in international investment law-making and the jurisprudence of investor-state dispute settlement tribunals involving the protection of the environment and human rights. As for law-making, this article explores the evolving substance of international investment agreements as well as regulatory developments in the area of business and human rights that are of relevance to the international investment law framework. The article then turns to consider the emergence of human rights and environmental issues in the recent jurisprudence of investment tribunals and appraises how such issues have been dealt with—both in procedural and substantive terms—by arbitral tribunals. Finally, it questions whether investment tribunals are appropriate venues for the adjudication of non-investment matters like environmental and human rights issues, and highlights best practices that could be adopted by future tribunals. Overall, the article concludes that the piecemeal approach adopted to date provides a step in the right direction but is ultimately inadequate given the multiple challenges that our planet currently faces. Rather, a more ambitious agenda that is concerned with promoting good investment, as opposed to mitigating bad practices, should be pursued.

*What’s the Use of a House if you Haven’t Got a Tolerable Planet to Put it On?* 1

## Introduction

This article appraises several recent developments in the area of foreign investment protection that suggest a greater sensitivity to environmental concerns and human rights. Classically, the latter non-economic interests have been perceived as irreconcilable with the promotion of foreign direct investment.^2^ However, this perception appears to be evolving as incremental changes in investment law-making and the adjudication of investor-state disputes have given greater weight to environmental and human rights issues.^3^ This resonates with a broader recognition at the international level that international corporations have a role to play in averting environmental damage and ensuring human rights are respected, evidenced not least in the United Nations Guiding Principles (UNGPs) and Sustainable Development Goals (SDGs).^4^

As such, these recent innovations in the international investment law framework are to be welcomed. That said, there is a danger that piecemeal development will result in too little, too late. In several respects, the developments examined in this article fall short of what is required given the threat that corporate activity can pose to the environment and human rights. While they appear to evidence a trend in the right direction, the character of reform must move beyond the enlightened view that environmental damage and human rights violations are negative externalities to be minimised. Rather, a more ambitious agenda must be pursued: the active promotion of green investment that enhances human welfare in every respect. Only this will do because, as we have seen in the starkest of terms through the recent COVID-19 pandemic, the very existence of corporate activity *depends* on good environmental governance and respect for human rights.

This article will first examine the evolving substance of international investment agreements (Sect. 2) before turning to related developments in the area of business and human rights that are of relevance to the international investment law framework (Sect. 3). Next, it considers the emergence of human rights and environmental issues in the recent jurisprudence of investment tribunals (Sect. 4), and asks whether investment tribunals are appropriate venues for the adjudication of non-investment matters like these (Sect. 5).

## International Investment Agreements, the Environment and Human Rights

A major underlying purpose of international investment agreements (IIAs) is commonly understood to be the protection of the investments of foreign investors and they are, primarily, a source of rights for such actors. While this remains the case, it is important to highlight several emerging exceptions to this. Indeed, a survey of a range of recent IIAs demonstrates that a greater number make provision for obligations incumbent upon investors.^5^ Alternatively, several other IIAs place further obligations on states to ensure that corporate actors respect the environment or human rights. These recent developments in treaty practice manifest themselves in a variety of ways.

The first example is in the preambular provisions of certain IIAs. In general, preambular provisions should reflect the object and purpose of a treaty. Typically, such IIA preambles set out the economic objectives that underlie the substantive provisions of the treaty, such as the promotion of reciprocal investment or mutually beneficial economic activity. Significantly for this analysis, several recent IIA preambles refer to environmental protection, labour rights or economic or sustainable development.^6^ Others explicitly recognize the connection that exists between investment and poverty reduction, job creation, or human development.^7^ That said, they do so while also retaining references to the more traditional objectives mentioned above.

The second notable area of development is in relation to the substantive provisions of such treaties. Several recent IIAs also contain specific provisions on respecting human rights, protecting the environment or promoting sustainable development, for example. Other provisions carve out space for the host state to respect obligations that exist under its domestic legal order, such as those that may concern the environment or human rights, as well as for the protection of public order and morals.^8^ Such provisions oblige investors to respect the law of the host state, public order and morals and have the effect of raising an obligation under a national legal regime from the domestic to the international level.^9^ Indeed, while the scope and formulation can vary significantly, a wide range of IIAs require investors to abide by the domestic law of the host state.^10^

Certain IIAs address foreign investors specifically by obliging them to respect human rights or ensure that they do not cause environmental damage.^11^ Such IIAs set out that investors should protect the environment around the location of their place of business and respect the human rights of their employees or others who may be affected by their activities, or operate their investments in a way that follows environmental, labour or human rights standards.^12^ Moreover, such IIAs may add that where there exists a difference between human rights or environmental standards in the home or the host state, the investor should follow whichever standards are higher.^13^ Alternatively, some IIAs commit investors to contribute to the economic, social and environmental development of the host state.^14^

Several IIAs oblige investors to undertake activities aimed at protecting the environment and human rights. For example, they may be required to conduct a social and/or environmental impact assessment of the investment before it is established.^15^ Moreover, investors may have to disseminate their environmental and social impact assessments in the local community or among other affected stakeholders.^16^ Further still, some IIAs require corporate actors to apply the precautionary principle, which emphasises caution where uncertainty about the damage caused by a project may exist, when making their environmental and social impact assessment.^17^

To ensure host states retain regulatory discretion in respect of environmental and human rights standards, and to avoid a race to the bottom in attracting investment from abroad, some IIAs encourage or oblige states against relaxing such standards. For example, IIAs can provide that states should not encourage investment by relaxing domestic health, safety or environmental measures and will oblige state parties to refrain from waiving or derogating from such measures.^18^ In a slightly different approach, other IIAs may encourage states to strive to continuously strengthen their environmental or human rights legislation.^19^

Some IIAs allow for states to regulate in certain non-economic areas, such as human rights or the environment, through general exceptions, much like Article XX of the General Agreement on Tariffs and Trade (GATT) in the context of international trade law.^20^ If measures adopted by the host state are ‘not applied in an arbitrary or unjustifiable manner’ nor ‘constitute a disguised restriction on international trade or investment’ they may be lawful as long as they are ‘necessary to protect human, animal or plant life or health’ or relate ‘to the conservation of living or non-living exhaustible natural resources’.^21^

IIAs increasingly refer to the corporate social responsibility of foreign investors.^22^ Moreover, such IIAs may provide that, if levels of corporate social responsibility rise over time, investors are expected to change their practices so as to adhere to the emerging higher levels.^23^ Other IIAs may refer to socially responsible practices or encourage corporate actors to implement policies that address labour standards, the environment, human rights, community relations or anti-corruption.^24^ These kinds of provisions in IIAs can serve as a conduit to bring wider international law norms and standards into the investment treaty arena. Indeed, there are a range of international instruments that are aimed at private actors to encourage them to respect human rights or protect the environment.^25^ In the context of foreign investment, the standards in such instruments may help to appraise how foreign investment may contribute to sustainable development, pose a risk to the environment or threaten respect for human rights.^26^ Tribunals may also measure the actions of private corporations against these standards whether or not there is explicit reference to them in the provisions of IIAs, as recent tribunal practice—explored below—demonstrates.

The application of the various substantive standards discussed above in international litigation requires certain procedural mechanisms, especially in view of the fact that, classically, dispute resolution under investment treaties envisages that an investor may bring a claim against a state, but not vice versa. Mechanisms to allow a state to bring a claim against an investor are, however, emerging in IIAs. Indeed, a relatively small number of IIAs contain provisions on counterclaims, which we will see below have been availed of by states to make certain non-economic claims in recent case law. Such express provision for counterclaims somewhat clarifies the uncertainty that had existed as to whether tribunals may hear these claims.^27^ Combined with explicit substantive provisions on human rights and environmental protection in IIAs, procedural mechanisms like these may help to provide a firmer legal basis for holding corporations accountable when they violate non-investment norms, including those concerning the environment or human rights.^28^ But there remain caveats, as their operation in practice shows.

## Related Developments in Business and Human Rights

Beyond the investment treaty-making context, a number of developments that have relevance for the international investment law framework are notable. In 2014 an intergovernmental working group was tasked with elaborating ‘an international legally binding instrument to regulate, in international human rights law, the activities of transnational corporations and other business enterprises’ in the form of a treaty on business and human rights.^29^ Early drafts suggest a preamble that calls upon businesses to ‘respect all human rights, including by avoiding causing or contributing to adverse human rights impacts through their own activities and addressing such impacts when they occur’.^30^ The substantive provisions of the treaty do encourage the strengthening of corporate liability under domestic law, including through procedural mechanisms. However, it seems unlikely that there would be direct corporate human rights obligations under the treaty.^31^

Separately, General Comment No. 24 of the Committee on Social, Economic and Cultural Rights was published in August 2017 on State obligations under the International Covenant on Economic, Social and Cultural Rights (ICESCR) in the context of business activities.^32^ Several aspects are noteworthy. First, in it the Committee directs that states consider whether any obligations under investment treaties they may enter into would conflict with those they are committed to under the ICESCR, and should refrain from joining such treaties where they find that there may indeed be a conflict.^33^ Second, it encourages states to conduct human rights impact assessments prior to entering into IIAs and to insert provisions that explicitly refer to their human rights commitments, as well as calling upon investor-state dispute settlement mechanisms to take human rights into consideration when they interpret the provisions of investment treaties.^34^ Third, the General Comment goes on to address the extra-territorial obligations of states in respect of the corporate sector and economic, social and cultural rights. In particular, the relevant part reminds states that their ‘obligations under the Covenant did not stop at their territorial borders’ and that they are ‘required to take the steps necessary to prevent human rights violations abroad by corporations domiciled in their territory and/or jurisdiction’ albeit ‘without infringing the sovereignty or diminishing the obligations of the host States under the Covenant’.^35^

In a different context again, the Hague Rules on Business and Human Rights Arbitration were published on 12 December 2019, along with commentaries to each of the provisions.^36^ Taking the 2013 UNCITRAL Arbitration Rules that govern the procedure of many arbitrations as a starting point, the Hague Rules have adapted the former to be more tailored to the matters that may be faced in business and human rights disputes. They have been developed over a five-year process, which included the involvement of a working group, a drafting team and a wide consultation process.^37^ The consultation process sought to garner input from business and arbitrators in particular. The project was led by a former judge of the International Court of Justice: Bruno Simma.

Through their substance, the Hague Rules have the potential to make an important contribution to the protection of human rights that may be implicated by corporate activity or the arbitration of corporate issues. Taking a comprehensive approach, they touch upon all aspects of business and human rights arbitration. First, as for the composition of the tribunal, they attempt to ensure that the presiding arbitrator has expertise relevant to the dispute^38^ and that the tribunal is diverse.^39^ Moreover, they provide for the appointment of emergency arbitrators who should be independent and impartial.^40^ Second, regarding arbitral procedure, the arbitral tribunal is obliged to conduct the proceedings in a ‘fair, efficient, culturally appropriate and rights-compatible’ way.^41^ Moreover, provision is made for more appropriate evidentiary procedures,^42^ and a strengthening of transparency and third-party participation, such as non-governmental organisations or states.^43^ Third party funding should be compulsorily disclosed, unless otherwise determined by the tribunal.^44^ Third, as for remedies, the Hague Rules give arbitral tribunals more power over interim measures^45^ and make provision for remedies that are better suited for business and human rights disputes.^46^

Significantly, the Hague Rules provide that the award should be compatible with human rights.^47^ The Commentary to this provision makes explicit that this duty incumbent on the arbitral tribunal is in part derived from Principle 31(f) of the UNGPs, which provides that non-judicial grievance mechanisms should be ‘rights compatible: ensuring that outcomes and remedies accord with internationally recognized human rights’.^48^ In this respect, while giving arbitral tribunals some discretion, the Commentary to the Hague Rules goes on to note that ‘it would be appropriate to include some discussion of the issue of rights-compatibility in the reasoning of any award’, although arbitral tribunals are cautioned against disregarding or altering the applicable law in the arbitration.^49^

Nevertheless, the Hague Rules will have to be consented to by parties if they are to be used in arbitration. Consent can be provided prior to a dispute arising, such as in a contract, or after a dispute has arisen, such as through an agreement between the parties.^50^ It is interesting to note that the Hague Rules place an emphasis on the resolution of disputes through negotiation or mediation, either prior to or during the arbitral proceedings.^51^ This may lead to more satisfactory and cost-effective solutions for the parties involved where disputes arise between them.

Overall, the Hague Rules on Business and Human Rights Arbitration attempt to redress the imbalance that can exist between parties in such litigation, as well as strive for compatibility between business and human rights. The next step will be for arbitral tribunals to recognize that a balance must be struck between economic interests and environmental or human rights interests. This is a step that *some* tribunals appear to be taking, as is evident from the appraisal of selected recent jurisprudence, which we turn to in the next section.

## Human Rights and Environmental Issues in Recent Jurisprudence of Investment Tribunals

### Human Rights in Investor-State Dispute Settlement

The most significant recent case involving human rights in investor-state dispute settlement is that of *Urbaser v. Argentina*,^52^ which concerned a concession for water distribution and sewage in Buenos Aires. This case involved a principal claim and a counterclaim, the character of each providing an opportunity for the Tribunal to consider the human rights of the affected population through its interpretation of the IIA provisions, drawing on the principle of systemic integration and referring to general principles of international law. That said, while the Tribunal placed an emphasis on the responsibility of corporate actors to respect human rights, it ultimately found in the counterclaim that the Claimant in this case was not obliged to ensure the right to water of the local population in Buenos Aires.

During the merits of the principal claim, the Tribunal took human rights into consideration when interpreting the relevant standard under the IIA at issue, in this case the fair and equitable treatment provision of the 1991 Spain-Argentina bilateral investment treaty. In the context of this principal claim, the Tribunal came to the view that Argentina was under a simultaneous obligation to fulfil both its investment law and its human rights law commitments.^53^ The Tribunal said that the state was obliged.

to ensure the population’s health and access to water and take all measures required to that effect. This was an important objective of the privatization of the water and sewage services […]. When measures had been taken that have as their purpose and effect to implement such fundamental rights […] they cannot hurt the fair and equitable treatment standard because their occurrence must have been deemed to be accepted by the investor when entering into the investment […]. In short, they were expected to be part of the investment’s legal framework.^54^

The need to ensure the fundamental rights of the local population, the Tribunal said, should have informed the investor’s legitimate expectations about how Argentina might have acted towards it when the investment was made.^55^ As a result, Argentina could not have acted unfairly or inequitably in breach of the relevant IIA that covered the investor’s investment in this sense.

The case is particularly notable, however, as it represents the first investment tribunal to accept jurisdiction over a counterclaim that concerns human rights. This procedural mechanism, as alluded to above, allows a host state to bring a claim against the investor, usually as long as the claim is in connection with the investment, in the context of a dispute that has been submitted by a foreign investor before an investor-state tribunal. In the *Urbaser* case, the Tribunal determined that there was a sufficient factual connection between the initial claim and the counterclaim. Argentina counterclaimed that Urbaser had breached its obligations under the concession contract to the extent that it did not adequately invest in the water distribution infrastructure and it was not possible to guarantee the right to water to the population in the areas concerned. The Tribunal noted that the claims and counterclaims were ‘based on the same investment, or alleged lack of sufficient investment, in relation to the same Concession’.^56^ This is a much more liberal approach than has been taken in previous awards, which have required a legal connection between the claim and counterclaim.^57^ Moreover, the Tribunal in *Urbaser* rejected the argument that it could not accept jurisdiction for a human rights claim.^58^ Further still, the Tribunal lowered the procedural burden by only requiring the host state to establish a *prima facie* case for jurisdiction.^59^

Interestingly, while deliberating on the merits of the counterclaim brought by Argentina against the investor for its failure to provide water to the population of Buenos Aires under the water concession it had been granted, the Tribunal was of the view that the IIA did not constitute a ‘closed system’.^60^ As a result, the state was entitled to invoke legal obligations beyond the treaty. The Tribunal also rejected the argument that the Claimant, being a non-state entity, could not be bound by human rights obligations. The Tribunal emphasized that human rights and labour standards were applicable to public and private actors, although it seemed to suggest that the character of commitment may be different for public and private actors respectively.

When considering the substance of the counterclaim, the Tribunal observed that ‘international law accepts corporate social responsibility as a standard of crucial importance for companies operating in the field of international commerce’.^61^ It also said that it can ‘no longer be admitted that companies operating internationally are immune from becoming subjects of international law’, although it further noted that certain soft law instruments were not ‘on their own sufficient to oblige corporations to put their policies in line with human rights law’.^62^ The Tribunal referred to the Universal Declaration of Human Rights as well as the ICESCR and the International Labour Organization’s (ILO) Tripartite Declaration of Principles and opined that they should be taken into consideration through Article 31(3)(c) of the Vienna Convention on the Law of Treaties (VCLT) in the interpretation of the IIA.^63^ This provision of the VCLT is also known as the principle of systemic integration and encompasses the notion that relevant rules of international law that apply in the relationship between the parties should be taken into account when interpreting a treaty.

The Tribunal ultimately went on to explain that, as regards the right to water, it ‘entails an obligation of compliance on the part of the state, but it does not contain an obligation for performance on part of any company providing a contractually required service’.^64^ Such an obligation was, in the Tribunal’s eyes, ‘distinct from the state’s responsibility to serve its population with drinking water and sewage services’.^65^ Unless the state had legislated to make such an obligation incumbent on private actors, the Tribunal was of the view that ensuring the right to water of local populations remained an obligation for the state to fulfil.^66^ As the Tribunal based its reasoning on human rights law via the reference to general principles of international law in the applicable law provision of the IIA, the result would likely have been different had the IIA referred explicitly to appropriate investor obligations or corporate social responsibility.

In the case of *Bear Creek v. Peru*, a tribunal decided that Peru had indirectly expropriated the investment of a Canadian mining company by revoking a licence granted to them as they sought to operate a silver mine in Peru.^67^ What is notable in this case, however, is a partial dissenting opinion by Arbitrator Philippe Sands. He was of the view that the behaviour of the investor, which led to social unrest in the local community around the investment, should have been taken into account in the determination of the compensation due to the investor. Referring to ILO Convention No. 169 concerning Indigenous and Tribal Peoples in Independent Countries, Sands considered that ‘the fact that the Convention may not impose obligations directly on a private foreign investor as such does not, however, mean that it is without significance or legal effects for them’.^68^ Sands also made reference to the *Urbaser* tribunal’s engagement with human rights considerations and observed that ‘[t]he same considerations apply in the present case in relation to the requirements of the ILO Convention 169, and in particular its Article 15 on consultation requirements’.^69^ He opined that the investor had not sought to obtain a social licence to operate as it failed to consult with the local population and establish trust. Consequently, he thought that the compensation awarded to the investor, Bear Creek, ought to have been mitigated in light of their contributory fault.

This case did not involve a counterclaim but nevertheless once again highlights that corporations are under increasing scrutiny for their compliance with international human rights norms. These more recent cases provide the strongest evidence yet of a trend that has been emerging in investment arbitration for over a decade. Indeed, several cases illustrate the increasing presence of human rights issues in investor-state dispute settlement cases, and arbitral tribunals have on several occasions recognized the importance that should be attached to human rights. In *Phoenix Action v. The Czech Republic*, for example, the Tribunal observed that ‘nobody would suggest that [investment] protection should be granted to investments made in violation of the most fundamental rules of protection of human rights, like investments made in pursuance of torture or genocide or in support of slavery or trafficking of human organs’.^70^ In *Spyridon Roussalis v. Romania*, a tribunal recognised that applicable obligations in investment arbitration ‘could include obligations deriving from multilateral instruments to which those states are parties, including, possibly, the European Convention of Human Rights and its Additional Protocol No. 1’.^71^

Human rights norms have also been interpreted in favour of investors in investment arbitration. In *Hesham T.M. Al Warraq v. Republic of Indonesia*, the Tribunal made extensive reference to various human rights instruments as it navigated its way through allegations of mistreatment in criminal proceedings conducted by the Indonesian authorities against the Claimant following a bank bailout.^72^ The Tribunal said that ‘the State Party undertakes to refrain from doing anything injurious to human rights and do everything to ensure respect for human rights of the individual person concerned. It is the failure to honour this obligation that amounts to a violation of the principles of good faith’.^73^ Ultimately, the Tribunal found that criminal proceedings violated the relevant standard in the IIA and, in interpreting this standard, it relied on the right to a fair trial under Article 14 of the International Covenant on Civil and Political Rights.^74^ That said, the Tribunal did not award damages in the case because the Claimant had breached the investor obligations clause in the IIA and, as a result, violated the clean hands doctrine.^75^ This also illustrates the role that investor obligations explicitly provided for in an IIA can play.

Similar developments are apparent in respect of international environmental norms in the investor-state dispute settlement arena, to which I will turn next.

### The Environment in Investor-State Dispute Settlement

Counterclaims have also been availed of in recent litigation involving environmental matters that have arisen in investor-state dispute settlement, and have even resulted in the first awards of compensation for environmental damage.^76^ In *Burlington Resources v. Ecuador*, a tribunal ordered the payment of $41 million to compensate environmental damage caused by the investor in that case.^77^ However, prior to this in 2012, the Tribunal had found that Ecuador had breached the relevant IIA for unlawfully expropriating Burlington’s investment, which consisted of oil production facilities in the Amazon rainforest, awarding the investor approximately $380 million.^78^

The Tribunal made the counterclaim award on the basis of Ecuadorian national law, which provided for strict liability where corporate actors caused environmental damage. Interestingly, the Tribunal appraised the damage that was caused by the investor and calculated the cost of reparation at each of the 40 sites in the oil field exploited by the Claimant and the Tribunal even made site visits.^79^

In a different case involving Ecuador, a tribunal handed down on 12 September 2014 a Decision on Remaining Issues of Jurisdiction and on Liability in *Perenco v. Ecuador*.^80^ There, the Tribunal was of the opinion that Ecuador was liable for breaches of fair and equitable treatment and expropriation under the applicable IIA. In 2015, the Tribunal issued an Interim Decision on the principal claims and the counterclaims that had subsequently been filed by Ecuador concerning the activities of Perenco.^81^

In the context of the counterclaim, Ecuador argued that the investor caused environmental damage by polluting parts of the Amazon rainforest. It submitted that it should be paid around $3 billion in compensation to repair the damage. Recognizing the difficulty in appraising the value of such damage and criticizing the testimony of the parties’ experts, the Tribunal in that case appointed its own independent environmental expert to assist with the task.^82^ On 27 September 2019, the Tribunal issued its final award.^83^ In this decision, the Tribunal decided on the damages that were to be apportioned in the case, awarding $449 million to Perenco and $54 million to Ecuador for its counterclaim.^84^

In its reasoning on the environmental counterclaim, the Tribunal noted that ‘[p]roper environmental stewardship has assumed great importance in today’s world’ and that if the relevant IIA permits it, the state may make a counterclaim for environmental damage caused by the investor and, if a tribunal is satisfied that the counterclaim is substantiated, the state should be awarded full reparation.^85^ The Tribunal in *Perenco v. Ecuador* explained that the Rio Declaration on Environment and Development had been an important source in drafting the Ecuadorian law on the protection of the environment^86^ and was of the view that ‘a State has wide latitude under international law to prescribe and adjust its environmental laws, standards and policies in response to changing views and a deeper understanding of the risks posed by various activities, including those of extractive industries such as oilfields’.^87^

Interestingly, Perenco had argued that Ecuador should pay any compensation awarded for the environmental counterclaim to an environmental remediation fund.^88^ Ecuador agreed to this.^89^ However, the Tribunal declined to make such an order.^90^ It considered that this ‘would require continued monitoring of Ecuador’s remediation activities’ and such monitoring ‘would be inconsistent with the Tribunal’s role’, not least because, following the issuance of the Award, ‘the Tribunal is *functus officio*’.^91^ That said, the Tribunal went on to declare its ‘firm expectation […] that the proceeds of the damages award made in favour of Ecuador in the environmental counterclaim will be devoted to remediation’.^92^

In *David Aven et al.* *v. Costa Rica*, a tribunal found that the Claimants had breached environmental law in Costa Rica and that Costa Rica was justified in interfering with a tourism project investment on the grounds of environmental protection.^93^ The Claimants submitted that they had received the required permits and approvals, including those related to the environmental viability of the project. However, following subsequent inspection of the site, Claimants argued that administrative and judicial actions were taken to shut down the project. According to Claimants, this destroyed the investment and breached the relevant IIA’s provisions on fair and equitable treatment, non-discrimination and expropriation.

Costa Rica responded that it took the measures it did to avoid environmental harm being caused to affected wetlands and forests. The state went on to argue that environmental protection was a legitimate policy, that environmental protection could be prioritized over the rights of investors and that it acted in accordance with domestic environmental law in order to protect its environment and ecosystems. Costa Rica also submitted a counterclaim against the Claimants for breach of the provisions on environmental protection under the IIA. In this context, Costa Rica asked the Tribunal to order that Claimants pay compensation to Costa Rica for repairing the environmental damage caused by Claimants’ activities.^94^

The Tribunal ultimately considered that Costa Rica had not violated the IIA.^95^ Moreover, it observed that the Claimants had caused environmental damage and that Costa Rica had acted to protect the wetland at risk, in accordance with domestic and international law. As for the environmental counterclaim filed by Costa Rica, the Tribunal rejected this for procedural reasons.^96^ Costa Rica had not properly presented its claim for environmental damage in a timely manner.^97^

That said, the Tribunal was of the view that ‘environmental law is integrated in many ways to international law, including [the applicable IIA]’^98^ and that states should implement appropriate legislation that would subject corporate actors to environmental obligations. Moreover, the Tribunal opined that investors were under an obligation to comply with measures taken at the national level for environmental protection and that there was no reason to excuse such actors from obligations addressed to them in IIAs like those at issue in the present case.^99^

By way of concluding this part, the above jurisprudence suggests that treaties having human rights and environmental obligations for investors is the most promising way to allow investment tribunals to hold corporate actors responsible for behaviour at odds with international standards. This will at the very least clarify for tribunals that they may take human rights or environmental obligations into account. It will also serve the purpose of clarifying that private actors too have a duty to respect such obligations. Other avenues that allow for corporate actors to be held to account for compliance with international human rights or environmental standards may be through domestic law or contractual obligations that become internationalized via an international investment agreement’s legality requirements or applicable law clause and the VCLT’s systemic integration provision.^100^

## Investment Tribunals as Appropriate Venues for Non-Investment Issues?

At this point we must ask whether investment tribunals are appropriate venues for the consideration of environmental or human rights norms? Arbitration has over a long time been the forum within which a wide variety of issues have been litigated, from territorial delimitation, the use of force, water rights and many others.^101^ We should not automatically assume that investor-state arbitration is necessarily an unsuitable forum for the consideration of non-investment issues and, as a flexible procedural mechanism, it has the potential to be adapted to the task at hand.

That said, it is important to be mindful of the risks. Indeed, it is imperative to ensure that those tribunals without the requisite expertise to decide cases involving complex matters of science around the environment or sensitive human rights issues do not do more harm than good. While many arbitrators who have distinguished investment law experience may not have such expertise in international environmental or human rights law, this is a deficiency that can be remedied. Among the recent jurisprudence I have discussed above and other cases, several tribunals have embraced innovative procedural safeguards in their adjudication of non-investment issues, which bodes well for the future.

Some investment tribunals have appointed their own experts to analyse scientific matters, as in *Perenco v. Ecuador* for example.^102^ In the latter case, the Tribunal recognized the difficulty in evaluating environmental damage and compensation on the basis of expert reports provided by the parties. As a result, the Tribunal appointed its own expert. The Tribunal also allowed the parties’ experts to comment on the reports of the Tribunal-appointed expert, bolstering the transparency of the approach adopted by the Tribunal.^103^ Interestingly, the parties were permitted to make two sets of written pleadings, oral pleadings as well as submit questions to the Tribunal-appointed expert during the hearings. Further still, during the hearings the party-appointed experts and counsel could hold witness conferences with the Tribunal-appointed expert.^104^ These kinds of safeguards help to ensure that legitimacy issues around the use of experts are addressed.^105^

The appointment of tribunal-appointed experts in international arbitration is, however, rare and has only occurred in a handful of cases.^106^ That said, outside the investor-state dispute settlement sphere the recourse to experts is becoming much more frequent, especially in cases involving the environment. In the *Pulp Mills on the River Uruguay* case, for example, the International Court of Justice (ICJ) noted that those submitting scientific or technical evidence should appear before the ICJ as experts rather than as counsel, which would allow them to be subjected to cross-examination.^107^ Scientific experts have since been resorted to in the *Whaling in the Antarctic* case,^108^ as well as in the *Construction of a Road in Costa Rica along the San Juan River*^109^ and in the *Maritime Delimitation in the Caribbean Sea and the Pacific Ocean* cases before the ICJ.^110^ Other than the ICJ, a tribunal constituted under Annex VII of the UN Convention on the Law of the Sea in the *South China Sea Arbitration Between the Republic of the Philippines and the People’s Republic of China* appointed its own *ex curia* experts so that the Tribunal could form its own ‘independent opinion’ on the environmental impact at issue.^111^

As an alternative or in addition to expert testimony, investment tribunals may allow for the submission of *amicus curiae* briefs as a way of obtaining informed opinion on non-economic interests beyond the economic issues at stake in a dispute, as was evident in the *Methanex v. USA*, *Suez v. Argentina* and *Biwater Gauff v. Tanzania* cases for example.^112^ Moreover, in *SAUR v. Argentina*, a human rights expert—Professor Christian Tomuschat—was appointed as an arbitrator.^113^ Further still, investment tribunals could engage with judicial, quasi-judicial or other bodies to develop norms and for guidance, as has been the case with the way in which the standard of fair and equitable treatment has been crafted in investor-state dispute settlement jurisprudence.^114^ Indeed, several compelling arguments have been made for more formal or informal dialogue between courts and tribunals and other institutions, particularly those institutions that are more specialized in human rights or environmental matters.^115^

## Concluding Remarks

These developments in international investment law suggest a greater appreciation for human rights and environmental matters. Indeed, the combination of explicit treaty provisions on human rights and the environment, evolving business and human rights instruments, the use of counterclaims, tools for the calculation of environmental damage, recourse to experts and *amicus curiae* all seem to be potentially effective ways of reconciling investment and non-investment issues. These are developments in the right direction for the field of international investment law, and should be welcomed. That said, in a landscape that comprises over 3000 IIAs^116^ and approximately 2000 known investor-state cases^117^ such developments represent merely green shoots in a typically barren world.

More can and must be done. In particular, law-makers should consider novel ways to incentivise green investment, promote responsibility among investors, encourage technology transfer and innovation, improve supply chains and ensure the broader implementation of good practices in the context of foreign direct investment to facilitate optimal environmental and human rights protection.^118^ Moreover, in dispute settlement, the practice with respect to the introduction of counterclaims could be clarified, experts and *amicus curiae* should be used more often and more consistently, innovative remedies might be conceived and arbitrators with expertise in environmental or human rights matters could be appointed.

Given the threats of climate change, pandemics, widespread inequality, poverty, and grave human rights abuse, corporate actors can in fact play a role in defeating these catastrophic phenomena. And so the green shoots explored in this article should be nurtured but, ultimately, large-scale forestation is required.

## Footnotes

1 Attributed to Henry David Thoreau. See Hudspeth (2013), p. 579.

2 For an explanation of this traditional perception, see Toral and Schultz (2010).

3 See, for example, Boisson de Chazournes (2017b); Simma (2011).

4 United Nations Guiding Principles on Business and Human Rights, endorsed by the Human Rights Council in HRC Resolution 17/4 on 16 June 2011, https://www.ohchr.org/documents/publications/guidingprinciplesbusinesshr_en.pdf (accessed 5 May 2020); UN General Assembly Resolution 70/1, ‘Transforming our world: The 2030 Agenda for Sustainable Development’, 25 September 2015, UN Doc. A/Res/70/1, particularly SDG 17.

5 See Boisson de Chazournes (2017a).

6 See, for example, Preamble, Treaty between the Government of the United States of America and the Government of the Republic of Rwanda Concerning the Encouragement and Reciprocal Protection of Investment, 19 February 2008; Preamble, Agreement Between the Government of Canada and the Government of the Republic of Benin for the Promotion and Reciprocal Protection of Investments, 9 January 2013; Preamble, Agreement Between the Government of Canada and the Government of the People’s Republic of China for the Promotion and Reciprocal Protection of Investments, 9 September 2012.

7 Preamble, Agreement for Cooperation and Investment Facilitation between Brazil and Mozambique, 30 March 2015.

8 Art. 9, Agreement on the Promotion, Protection and Guarantee of Investments among Member States of the Organization of the Islamic Conference, 1 June 1981.

9 See, for example, *Hesham T. M. Al Warraq v. Republic of Indonesia*, Award, UNCITRAL, 15 December 2014, para. 663.

10 See, for example, Art. 13, Investment Agreement in the Common Market for Eastern and Southern Africa (COMESA) Area, 23 May 2007, which requires that ‘COMESA investors and their investments shall comply with all applicable domestic measures of the Member State in which their investment is made’; Art. 11, Southern African Development Community (SADC) Model Bilateral Investment Treaty (BIT), July 2012, which is narrower in its coverage of domestic law, requires that: ‘Investors and Investments shall comply with all laws, regulations, administrative guidelines and policies of the Host State concerning the establishment, acquisition, management, operation and disposition of investments’.

11 See, for example, Art. 18(2), Reciprocal Investment Promotion and Protection Agreement between the Government of the Kingdom of Morocco and the Government of the Federal Republic of Nigeria, 3 December 2016; Art. 14(2), Supplementary Act A/SA.3/12/08 Adopting Community Rules on Investment and the Modalities for their Implementation with ECOWAS, 19 December 2008; Arts. 13 and 15, SADC Model BIT, 2012; Art. 24, Draft Pan-African Investment Code, 2016; Art. 12, India Model Bilateral Investment Treaty (BIT), 2016. On the impact of the Morocco-Nigeria BIT, 2016, see Gazzini (2017).

12 See, for example, Arts. 13, 14, 15, 16, and 22 SADC Model BIT, 2012; Arts. 19(2)-(3), 20(1), 22, 24, and 37(3), Draft Pan-African Investment Code; Art. 12(1)-(2), India Model BIT, 2016.

13 See, for example, Art. 15(3), SADC Model BIT, 2012.

14 See, for example, Art. 22(3), Draft Pan-African Investment Code, 2016.

15 See, for example, Art. 14(2), Morocco-Nigeria BIT, 2016; Art. 12, Supplementary Act A/SA.3/12/08 Adopting Community Rules on Investment and the Modalities for their Implementation with ECOWAS, 19 December 2008.

16 Art. 12, Supplementary Act A/SA.3/12/08 Adopting Community Rules on Investment and the Modalities for their Implementation with ECOWAS, 19 December 2008.

17 Ibid.

18 Art. 3, Protocol of the Agreement between the Swiss Confederation and the United Mexican States on the Promotion and Reciprocal Protection of Investments, 10 July 1995; Art. 7, Agreement between the Belgium-Luxembourg Economic Union and the Government of the Republic of Mozambique on the Reciprocal Promotion and Protection of Investment, 18 July 2006.

19 Art. 7, Agreement between the Belgium-Luxembourg Economic Union and the Government of the Republic of Mozambique on the Reciprocal Promotion and Protection of Investment, 18 July 2006.

20 General Agreement on Tariffs and Trade, 55 UNTS 187, 1 January 1948.

21 See, for example, Art. 33, Agreement Between the Government of Canada and the Government of the People’s Republic of China for the Promotion and Reciprocal Protection of Investments, 9 September 2012; Art. IX, Agreement between Canada and the Slovak Republic for the Promotion and the Protection of Investments, 20 July 2010; Art. XVII-2, Agreement between the Government of Romania and the Government of Canada for the Promotion and Reciprocal Protection of Investments, 8 May 2009.

22 See, for example, Art. 10, Agreement for Cooperation and Investment Facilitation between Brazil and Mozambique, 30 March 2015; Art. 16, Agreement Between the Government of Canada and the Government of the Republic of Benin for the Promotion and Reciprocal Protection of Investments, 9 January 2013. See also, Art. 7, The Netherlands Model BIT, 2019. For an interesting examination of the latter, see Duggal and van de Ven (2019)*.*

23 See, for example, Chapter III on the Obligations and Duties of Investors and Investments, Supplementary Act A/SA.3/12/08 Adopting Community Rules on Investment and the Modalities for their Implementation with ECOWAS, 19 December 2008.

24 See, for example, Art. 9, Investment Cooperation and Facilitation Agreement between Brazil and Malawi, 25 June 2016; Art. 16, Agreement Between the Government of Canada and the Government of the Republic of Benin for the Promotion and Reciprocal Protection of Investments, 9 January 2013.

25 United Nations Global Compact (2000); International Labour Organization’s Tripartite Declaration of Principles concerning Multinational Enterprises and Social Policy (1977, amended 2000/2006/2017); OECD Guidelines for Multinational Enterprises (2011); and UNGPs. Similarly, the SDGs, which have been endorsed by UN Member States as well as actors in the private sector, are relevant to private actors.

26 Fariello et al. (2016), p. 194; Krajewski (2018).

27 Art. 28(9), COMESA Investment Agreement provides a clear example and sets out that ‘a Member State against whom a claim is brought by a COMESA investor […] may assert as a defense, counterclaim, right of set off or other similar claim, that the COMESA investor bringing the claim has not fulfilled its obligations’. Arts. 19 and 29(19), SADC Model BIT make similar provision, as does Art. 43, Draft Pan-African Investment Code.

28 Krajewski (2018).

29 UN Human Rights Council, Resolution 26/9 (2014), para. 1.

30 Bernaz (2018).

31 Ibid.

32 Committee on Economic, Social and Cultural Rights, General Comment No. 24 (2017) on State Obligations under the International Covenant on Economic, Social and Cultural Rights in the Context of Business Activities, 10 August 2017, E/C.12/GC/24.

33 Ibid., para. 13.

34 Ibid.

35 Ibid., para. 26.

36 Hague Rules on Business and Rights Arbitration, 12 December 2019, https://www.cilc.nl/cms/wp-content/uploads/2019/12/The-Hague-Rules-on-Business-and-Human-Rights-Arbitration_CILC-digital-version.pdf (accessed 5 May 2020).

37 Foreword, ibid.

38 Art. 11(1)(c), ibid.

39 Art. 11(3), ibid.

40 Art. 31, ibid.

41 Art. 18, ibid.

42 Arts. 27, 28 and 30(3), ibid.

43 Arts. 24 bis and 33–38, ibid.

44 Art. 55, ibid.

45 Art. 26, ibid.

46 Art. 40, ibid.

47 Art. 45(4), ibid.

48 Principle 31(f), UNGPs.

49 Commentary to Art. 45, Hague Rules on Business and Human Rights Arbitration.

50 Introductory note, ibid.

51 See, for example, Art. 56, ibid.

52 *Urbaser SA and Consorcio de Aguas Bilbao Bizkaia, Bilbao Biskaia Ur Partzuergoa v. The Argentine Republic*, Award, ICSID Case No. ARB/07/26, 8 December 2016.

53 Ibid., para. 720.

54 Ibid., para. 622.

55 Ibid., para. 624.

56 Ibid., para. 1151.

57 See, for example, *Saluka Investments BV v. The Czech Republic*, Decision on Jurisdiction over the Czech Republic’s Counterclaim, UNCITRAL, 7 May 2004.

58 *Urbaser SA and Consorcio de Aguas Bilbao Bizkaia, Bilbao Biskaia Ur Partzuergoa v. The Argentine Republic*, Award, ICSID Case No. ARB/07/26, 8 December 2016, para. 1154.

59 Ibid., para. 1153.

60 Ibid., para. 1191.

61 Ibid., para. 1195.

62 Ibid.

63 Ibid., paras. 1198, 1200–1203. For an insightful analysis of the Tribunal’s reasoning on this point, see de Brabandere (2018).

64 *Urbaser SA and Consorcio de Aguas Bilbao Bizkaia, Bilbao Biskaia Ur Partzuergoa v. The Argentine Republic*, Award, ICSID Case No. ARB/07/26, 8 December 2016, para. 1208.

65 Ibid.

66 Ibid., para. 1213.

67 *Bear Creek Mining v. Peru*, Award, ICSID Case No. ARB/14/21, 30 November 2017.

68 Ibid., Partial Dissenting Opinion of Philippe Sands, para. 10.

69 Ibid., para. 11.

70 *Phoenix Action v. The Czech Republic*, Award, ICSID Case No. ARB/06/5, 15 April 2009, para. 78.

71 *Spyridon Roussalis v. Romania*, ICSID Case No. ARB/06/1, Award, 7 December 2011, para. 312.

72 *Hesham T.M. Al Warraq v. Republic of Indonesia*, UNCITRAL, Award, 15 December 2014.

73 Ibid., para. 560.

74 Ibid., paras. 556 et seq.

75 Ibid., para. 654.

76 *Burlington Resources v. Ecuador*, Decision on Ecuador’s Counterclaim, ICSID Case No. ARB/08/5, 7 February 2017; *Perenco v. Ecuador and Empresa Estatal Petróleos del Ecuador (Petroecuador)*, Interim Decision on the Environmental Counterclaim, ICSID Case No. ARB/08/6, 11 August 2015; *Perenco Ecuador Ltd. v. The Republic of Ecuador and Empresa Estatal Petróleos del Eduador (Petroecuador)*, Final Award, ICSID Case No. ARB/08/6, 27 September 2019. For a comprehensive analysis of this topic, see Rudall (2020); Rudall (2018).

77 *Burlington Resources v. Ecuador*, Decision on Ecuador’s Counterclaim, ICSID Case No. ARB/08/5, 7 February 2017.

78 *Burlington Resources v. Ecuador*, Decision on Liability, ICSID Case No. ARB/08/5, 14 December 2012; *Burlington Resources v. Ecuador*, Decision on Reconsideration and Award, ICSID Case No. ARB/08/5, 7 February 2017.

79 *Burlington Resources v. Ecuador*, Decision on Ecuador’s Counterclaim, ICSID Case No. ARB/08/5, 7 February 2017, para. 18.

80 *Perenco Ecuador Ltd. v. The Republic of Ecuador and Empresa Estatal Petróleos del Eduador (Petroecuador)*, Decision on Remaining Issues of Jurisdiction and on Liability, ICSID Case No. ARB/08/6, 12 September 2014.

81 *Perenco Ecuador Ltd. v. The Republic of Ecuador and Empresa Estatal Petróleos del Eduador (Petroecuador)*, Interim Decision on the Environmental Counterclaim, ICSID Case No. ARB/08/6, 11 August 2015.

82 Ibid., para. 587.

83 *Perenco Ecuador Ltd. v. The Republic of Ecuador and Empresa Estatal Petróleos del Eduador (Petroecuador)*, Final Award, ICSID Case No. ARB/08/6, 27 September 2019.

84 Ibid.

85 Ibid., para. 34.

86 Ibid., para. 331.

87 Ibid., para. 35.

88 Perenco’s Comments to the Independent Expert Report dated 22 February 2019, ICSID Case No. ARB/08/6, para. 75.

89 *Perenco Ecuador Ltd. v. The Republic of Ecuador and Empresa Estatal Petróleos del Eduador (Petroecuador)*, Final Award, ICSID Case No. ARB/08/6, 27 September 2019, paras. 900–901.

90 Ibid., para. 902.

91 Ibid.

92 Ibid., para. 904.

93 *David Aven **et al.* *v. Costa Rica,* Award, DR-CAFTA Case No. UNCT/15/3, 18 September 2018.

94 Ibid., para. 715.

95 Ibid., para. 585.

96 Ibid., para. 742.

97 Ibid., para. 745.

98 Ibid., para. 737.

99 Ibid., para. 739.

100 De Brabandere (2018).

101 Desierto (2019).

102 *Perenco Ecuador Ltd. v. The Republic of Ecuador and Empresa Estatal Petróleos del Eduador (Petroecuador)*, Interim Decision on the Environmental Counterclaim, ICSID Case No. ARB/08/6, 11 August 2015.

103 Ibid., para. 588.

104 Ibid. See also *Perenco Ecuador Ltd. v. The Republic of Ecuador and Empresa Estatal Petróleos del Eduador (Petroecuador)*, Final Award, ICSID Case No. ARB/08/6, 27 September 2019, paras. 627–630.

105 Mbengue (2015); Boisson de Chazournes et al. (2018).

106 See Páez-Salgado and Zuleta (2019); Choudhury (2018).

107 *Pulp Mills on the River Uruguay (Argentina v. Uruguay)*, ICJ Reports 2010, p. 14, para. 167.

108 *Whaling in the Antarctic (Australia v. Japan)*, ICJ Reports 2014, p. 226, paras. 74–75.

109 *Construction of a Road in Costa Rica along the San Juan River (Nicaragua v. Costa Rica)*, Judgment, 16 December 2015, paras. 45, 175–176, 204.

110 *Maritime Delimitation in the Caribbean Sea and the Pacific Ocean (Costa Rica v. Nicaragua) / Land Boundary in the Northern Part of Isla Portillos (Costa Rica v. Nicaragua),* General List Nos. 157 and 165, 2 February 2018, paras. 14, 71, 73, and 86.

111 *South China Sea Arbitration Between the Republic of the Philippines and the People’s Republic of China*, Award, paras. 84, 136 and 821.

112 See, for example, *Methanex v. USA*, Decision of the Tribunal on Petitions from Third Persons to Intervene as Amici Curiae, NAFTA, 15 January 2001; *Suez v. Argentina*, Order in Response to a Petition for Transparency and Participation as Amicus Curiae, ICSID Case No. ARB/03/19, 19 May 2005; *Biwater Gauff v. Tanzania*, Petition for Amicus Curiae Status, ICSID Case No. ARB/05/22, 27 November 2006.

113 *SUAR v. Argentina*, Award, ICSID Case No. ARB/04/4, 22 May 2014.

114 Teitel and Howse (2008), p. 989.

115 Ibid., p. 981; On judicial dialogue and comity more generally, see Slaughter (1994); Slaughter (2000), pp. 1112–1115.

116 UNCTAD IIA Navigator, https://investmentpolicy.unctad.org/international-investment-agreements (accessed 5 May 2020).

117 UNCTAD Investor State Dispute Settlement Navigator, https://investmentpolicy.unctad.org/investment-dispute-settlement (accessed 5 May 2020).

118 Magraw and Puig (2018), p. 2718.
